# Alveolar dynamics during mechanical ventilation in the healthy and injured lung

**DOI:** 10.1186/s40635-019-0226-5

**Published:** 2019-07-25

**Authors:** Jana Grune, Arata Tabuchi, Wolfgang M. Kuebler

**Affiliations:** 1 Institute of Physiology, Charité-Universitätsmedizin Berlin, Charitéplatz 1, 10117 Berlin, Germany; 2 DZHK (German Centre for Cardiovascular Research), partner site Berlin, 10117 Berlin, Germany; 3 The Keenan Research Centre for Biomedical Science at St. Michael’s, Toronto, Canada; 4 Departments of Surgery and Physiology, University of Toronto, Toronto, Canada

**Keywords:** Mechanical ventilation, Alveolar dynamics, PEEP, Lung physiology, ARDS, Opening/collapse

## Abstract

Mechanical ventilation is a life-saving therapy in patients with acute respiratory distress syndrome (ARDS). However, mechanical ventilation itself causes severe co-morbidities in that it can trigger ventilator-associated lung injury (VALI) in humans or ventilator-induced lung injury (VILI) in experimental animal models. Therefore, optimization of ventilation strategies is paramount for the effective therapy of critical care patients. A major problem in the stratification of critical care patients for personalized ventilation settings, but even more so for our overall understanding of VILI, lies in our limited insight into the effects of mechanical ventilation at the actual site of injury, i.e., the alveolar unit. Unfortunately, global lung mechanics provide for a poor surrogate of alveolar dynamics and methods for the in-depth analysis of alveolar dynamics on the level of individual alveoli are sparse and afflicted by important limitations. With alveolar dynamics in the intact lung remaining largely a “black box,” our insight into the mechanisms of VALI and VILI and the effectiveness of optimized ventilation strategies is confined to indirect parameters and endpoints of lung injury and mortality.

In the present review, we discuss emerging concepts of alveolar dynamics including alveolar expansion/contraction, stability/instability, and opening/collapse. Many of these concepts remain still controversial, in part due to limitations of the different methodologies applied. We therefore preface our review with an overview of existing technologies and approaches for the analysis of alveolar dynamics, highlighting their individual strengths and limitations which may provide for a better appreciation of the sometimes diverging findings and interpretations. Joint efforts combining key technologies in identical models to overcome the limitations inherent to individual methodologies are needed not only to provide conclusive insights into lung physiology and alveolar dynamics, but ultimately to guide critical care patient therapy.

## Background

Despite the critical impact of alveolar dynamics on respiratory function, gas exchange, and lung stability, simple key determinants such as changes in alveolar size, shape, and number of recruited alveoli during the breathing cycle remain poorly understood. Emerging concepts of alveolar dynamics including alveolar expansion/contraction, stability/instability, and opening/collapse remain controversial and, in part, mutually exclusive, but are at the same time fundamental for the understanding of alveolar-based diseases and the optimization of ventilation strategies in critical care. Based on experimental and clinical data obtained by state-of-the-art imaging techniques, we review current concepts of alveolar dynamics in intact and diseased lungs and discuss their potential clinical impact.

## The black box of alveolar dynamics in mechanical ventilation

Mechanical ventilation is a life-saving therapy in critical care patients; yet, mechanical ventilation may by itself cause severe co-morbidities in that it can trigger ventilator-associated lung injury (VALI) in humans or ventilator-induced lung injury (VILI) in experimental animal models [1], respectively, as well as neurological deficits [2], muscle wasting [3], and ultimately systemic multi-organ failure [4]. The detrimental effects of mechanical ventilation with high inspiratory pressures or high tidal volumes were first documented in small clinical trials [5] and animal experiments [6] and were ultimately confirmed in 861 patients in the eminent ARDS network trial which demonstrated a 22% reduction in mortality in patients with the acute respiratory distress syndrome (ARDS) ventilated with lung-protective tidal volumes of 6 mL/kg body weight (bw) as compared to the then standard of 12 mL/kg bw [7]. Since then, the widespread adoption of ventilation strategies with lower tidal volumes [8–10] has led to a marked reduction in mortality rates in ARDS patients, although in-hospital mortality still remains as high as 30% [11].

A series of different biomechanical mechanisms have been proposed to cause or contribute to VALI/VILI, including barotrauma (i.e., alveolar air leak due to excessive ventilation pressures), volutrauma (i.e., overdistension by high tidal volumes with stress failure of endothelial and/or epithelial barrier function), atelectrauma (i.e., cyclic recruitment/derecruitment of alveolar units open during inspiration and collapsed during expiration) [12], and biotrauma (i.e., release of inflammatory mediators by stretch-activated mechanotransduction signaling cascades in the lung) [13], as well as ergotrauma (i.e., energy expansion by the dynamics of high stress respiratory cycles) [14], vascular trauma (endothelial injury due to alveolar expansion) [15–21], and hemodynamic trauma (endothelial injury due to increased capillary hydrostatic pressures or shear forces, respectively) [22, 23]. Based on these different concepts, a range of ventilation strategies have been proposed, tested, and partially implemented to prevent or attenuate the detrimental consequences of mechanical ventilation, including low tidal volume ventilation, high positive end-expiratory pressures (PEEP), sighs and recruitment maneuvers, high-frequency oscillatory ventilation, and noisy ventilation. However, except for low tidal volume ventilation [7] and prone positioning in patients with severe ARDS [24], none of these modifications yielded significant benefit in terms of patient mortality in multi-centric, randomized clinical trials. It seems fair to speculate that the failure of many trials may be attributable to the fact that not all patients with ARDS will benefit from identical ventilator settings;in other words, that mechanical ventilation should be personalized [25]. This notion is highlighted by the recent identification of at least two different ARDS subphenotypes by latent class analyses which diverge markedly with respect to their response to high PEEP ventilation in that patients with a hyperinflammatory phenotype showed a decrease in mortality when ventilated with high PEEP, whereas mortality increased with PEEP in patients with a less inflammatory subphenotype [26].

A key inherent problem in the stratification of patients for personalized ventilation, but even more so for our overall understanding of VILI and the conceptual development, testing, and implementation of optimized ventilation strategies, lies in our limited insight into the effects of mechanical ventilation at the actual site of injury, i.e., the alveolar unit. Unfortunately, global lung mechanics provide for a poor surrogate of alveolar dynamics, e.g., in that inflection points on the P-V curve do not correlate with alveolar recruitment [27]. The extent of this “black box” becomes already evident at the level of static lung mechanics in that our knowledge of the actual stretch effects during mechanical ventilation is in fact very rudimentary. Since the 1999 landmark study by Daniel Tschumperlin and Susan Margulies which showed that the epithelial basement membrane surface area of the rat lung increases by 7.5 and 33.6% when the lungs expand from 42 to 100% of total lung capacity [28], these values have become the benchmark for in vitro studies addressing the effects of mechanical stretch on the alveolar epithelium. While such standards are unquestionably useful, it is important to keep in mind that they present a gross oversimplification of the actual stretch effects in the intact lung, which are—among other things—locally affected by the complex three-dimensional (3D) structure of the alveoli with individual alveolar epithelial cells simultaneously outlining adjacent alveolar structures [29], at the regional level by marked heterogeneities in alveolar inflation as exemplified by the baby lung concept according to which some lung areas overinflate while others remain stiff or collapsed [30] and at the global lung level by volume-dependent stretch effects of extra-alveolar airspaces [31, 32]. Likewise, the indiscriminating adoption of these epithelial stretch values for other pulmonary cells like endothelial cells or fibroblasts must be regarded with a huge grain of salt—we simply do not know the extent of stretch these cells undergo. The problem becomes exponentially amplified when we move from static lung mechanics to dynamic alveolar kinetics in healthy and injured lungs.

With alveolar dynamics in the intact lung remaining largely a “black box,” our insight into the mechanisms of VILI and the effectiveness of specific ventilation strategies is confined to indirect longitudinal parameters and endpoints of lung injury and mortality. Conversely, better tools to study ventilation dynamics at the alveolar level and the resulting mechanistic and therapeutic insight can be expected to pave the way for both the identification of novel pharmacological targets and the optimization of mechanical ventilation strategies. In the present review, we discuss emerging concepts of alveolar dynamics including alveolar expansion/contraction, stability/instability, and opening/collapse. Many of these concepts remain controversial, to a considerable degree due to different methodologies applied. We therefore preface our review with an overview of existing technologies and approaches for the analysis of alveolar dynamics, highlighting their individual strengths and limitations which may provide for a better appreciation of the sometimes diverging findings and interpretations.

## Analysis of alveolar dynamics

### Histology and electron microscopy

Less than 100 years ago, in 1929, the eminent physiologist Charles Macklin still proposed that a muscle system running from the larynx to the alveolar mouths would cyclically shorten and narrow the alveolar ducts forcing out the air flow [33]. Only 30 years later, however, the view on alveolar dynamics had dramatically changed thanks to comprehensive (albeit static) investigations into pulmonary physiology by histological methods [34–38]. In 1962, Storey and Staub developed a rapid-freeze method for immediate conservation of mechanically ventilated feline lungs at different time points of the respiratory cycle, enabling them to investigate the lung at different lung volumes [39]. From stereomicroscopic measurements of 3D histological sections, the authors calculated an increase of alveolar surface area of 70% when the lung volume is increased from the functional residual capacity to 75–80% of total lung capacity, thereby providing first proof for the concept of alveolar expansion during inspiration. The use of electron microscopy for the analysis of histological lung sections further paved the way for the in-depth analysis of the effects of mechanical ventilation at the alveolar level [40]. From their studies in ventilated rabbits, Gil and coworkers proposed four conceivable mechanisms by which lung volume may change during inflation and deflation, namely (1) sequential recruitment–decruitment of alveoli, (2) isotropic “balloon-like” alveolar volume (VA) change, (3) simultaneous changes in alveolar size and shape, and (4) crumpling and decrumpling of the alveolar surface [40]. Despite the significant advances by electron microscopic studies in the visualization and quantitative analysis of the alveolar surface, this approach remained limited to the morphological and morphometric assessment of fixed lung tissue. In consequence, it lacks the power to resolve dynamic processes as highlighted by its inability to differentiate between the proposed mechanisms of lung volume change.

### Intravital microscopy

Intravital microscopy (IVM) of the lung for real-time visualization of pulmonary macro- and microhemodynamics had been carried out as early as 1930, but the approach received a major boost through the introduction of implantable thoracic windows in the dog by Wiltz Wagner and coworkers [41] and a subsequent adaptation to the rabbit by our group [42]. The latter models utilized windows with suction manifolds allowing to partially immobilize the lung thus providing sufficient stabilization of the area of interest for live microscopy. While these approaches were primarily utilized for the study of capillary recruitment and leukocyte kinetics in the lung, proof-of-principle studies hinted at their potential for the study of alveolar dynamics [43].

Starting in the late 1990s by work from Gary Nieman’s group, IVM has increasingly been used for the study of lung volume changes at the alveolar level in vivo [44]. In anesthetized, intubated, and ventilated mongrel dogs, Carney and colleagues obtained direct microscopic access to sub-pleural alveoli via a thoracotomy through the left fourth intercostal space [44]. From these studies, the authors suggested that volume changes in the healthy intact lung are primarily the result of cyclic recruitment/decruitment of individual alveoli rather than alveolar expansion, a concept that has since remained a topic of controversy in the field [45] due to the potential for non-physiological effects introduced by surgical approaches, lung immobilization, or microscopy, as well as pitfalls in image analysis and interpretation which are inherent to lung IVM.

Over the past two decades, IVM techniques have advanced further in terms of closed chest conditions without direct application of mechanical traction forces on the observation area and the use of mice as species of choice [46–53]. To minimize mechanical impacts on alveolar dynamics, our own lab developed an IVM technique for mice which avoids immobilization of the lung surface by suction devices or cover slips but rather simulates the situation of a freely moving lung under closed chest conditions following implantation of a transparent thoracic window into the right chest wall [48]. This technique allows for real-time observations under closed chest and physiological pressure conditions, however, at the expense of continuous tissue movements which can be partially compensated by triggering image acquisition to the inspiratory and/or expiratory plateau phases. Conversely, Looney and colleagues developed a model for two-photon microscopic studies in mice in which the observation area is stabilized via a metal vacuum chamber, facilitating continuous cell tracking yet in parallel impairing alveolar dynamics [46].

Despite these persisting limitations in terms of discontinuous visualization of areas-of-interest vs. mechanical fixation and resulting impairments in alveolar dynamics, IVM remains the methodological gold standard for the assessment of alveolar dynamics due to its high temporal and spatial resolution and real-time assessment of alveolar physiology. The importance of IVM for our current understanding of lung physiology and disease is exemplified by a series of hallmark papers challenging existing paradigms and introducing new concepts such as pre-capillary oxygen uptake in the lung, the concept of alveolar pendelluft, and the transport of T cells and neutrophils in pulmonary capillaries [46, 49, 50, 54, 55].

### Optical coherence tomography

Despite its unmatched advantages in temporo-spatial resolution, IVM also comes along with several major limitations, in that visualization is commonly limited to two-dimensional imaging, is largely confined to the analysis of subpleural alveoli, and may suffer from image artifacts caused by light reflection at the alveolar air-liquid interface [56]. To overcome some of these limitations, optical coherence tomography (OCT) has been utilized to provide 3D information of alveolar shape and size in vivo [56, 57]. OCT is an interferometric, optical imaging technique, allowing the acquisition of microscopic-resolution snap-shots of subpleural alveoli [57–59]. OCT-derived 3D images are generated up to a depth of 500 μm of subpleural lung regions with a resolution of 10 μm [57]. Therefore, lung tissue can be visualized in defined spatial planes beneath the surface regions, enabling the investigation of deeper lung areas and post hoc 3D image reconstruction.

From a practical point of view, IVM and OCT imaging share a number of similarities in that both techniques require surgical procedures to provide visual access for the visualization of subpleural alveoli, limiting the utilization of both techniques to preclinical animal models. Notably, endoscopic confocal imaging techniques originally developed for imaging of bronchial walls also allow for visualization of alveoli in vivo [60], yet quantitative analysis of alveolar dynamics from these recordings is precluded by the continuous shift of alveoli in and out of the focus plane (own observation). Analogous to IVM, we and others have used OCT successfully to visualize alveolar dynamics during lung inflation and deflation [57, 58, 61, 62]. The technique provides 3D data sets, but has limited spatial resolution [56]. Due to its tomographic character, image acquisition has to be triggered to mechanical ventilation, with each 3D image being recorded over a series of subsequent respiratory cycles, preventing continuous real-time imaging of alveolar dynamics. However, ventilation-dependent alveolar dynamics can be reconstructed off-line from 3D data sets acquired at different time points of the respiratory cycle.

**Fig. 1:**
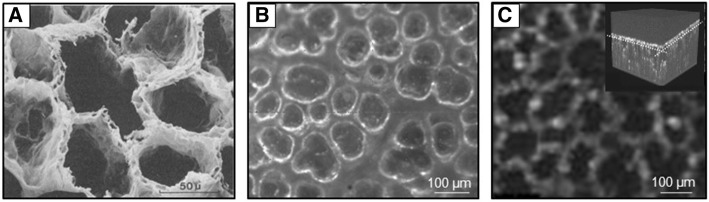
**a** Electron micrograph of air-filled lungs fixed by vascular perfusion [40] **b** Representative darkfield IVM image of a healthy murine lung [59] **c** Representative OCT image of subpleural alveolar lung parenchyma of an isolated rabbit lung from a 3D image stack of 0.8 × 0.8 × 0.4-mm size [57]

Despite its lower spatial and temporal resolution, OCT imaging offers some unique additional advantages over IVM imaging in excess of its ability for 3D imaging. In IVM, oblique dark field illumination setups used for lung imaging commonly comprise a ring light source to illuminate the lung surface while excluding excessive light reflexes from the pleural surface. Light reflection and refraction of this oblique illumination at the alveolar air-liquid interface results in two circular reflexes for each individual alveolus termed a double-ring structure [56]. The outer circular reflex is widely used to discriminate between alveolar lumen and surrounding tissue [31, 63, 64]; however, due to complex light reflection and refraction in the heterogeneous tissue comprised of air-filled alveoli imbedded in a lung parenchyma, it is still partly unclear to which extent this reflex indeed yields a quantitatively accurate depiction of the alveolar wall. Moreover, in contrast to OCT imaging, IVM is not able to distinguish partially fluid-filled from collapsed alveoli as both phenomena will result in a loss of the circular light reflex outlining the alveolar structure. This effect was elegantly demonstrated by Gaertner and coworkers in a direct comparison of IVM data from alveoli in a mouse model with simultaneously acquired OCT images [56]. As we will discuss later, the lacking ability of IVM to discriminate between collapsed and fluid-filled alveoli may have contributed significantly to some of the ongoing controversies regarding alveolar dynamics in mechanically ventilated lungs. Importantly, the individual strengths of IVM (real-time, high spatial, and temporal resolution) and OCT (3D imaging, tissue penetration, differentiation between collapsed, and fluid-filled alveoli) complement each other synergistically to overcome the limitations inherent to each individual technique (Fig. 1).

### Computed tomography

In patients, the use of computed tomography (CT) has become the routine method of choice to address respiratory morphology during mechanical ventilation [65]. CT imaging was first applied in ARDS patients in the mid 1980s as a tool to enhance the in-depth understanding of the pathophysiology of the disease [66, 67]. For example, large CT-based clinical trials revealed the role of lung volume reduction surgery and the response to PEEP in ARDS patients as predictors of outcome [68, 69]. Specifically, the studies by Gattinoni and coworkers have substantially advanced and shaped our present understanding of ARDS [70–72]. Their studies demonstrate that large areas of an injured lung are derecruited at low PEEP and can only partially be recruited by high PEEP ventilation, a recognition which fueled the development of the “baby lung” concept [30]. Over and above that, a series of CT-based studies from the ARDS study group has addressed the critical prior knowledge gap regarding the distribution of aerated and nonaerated lung regions in ARDS patients [73–75]. In these studies, the authors measured dimensions of the lungs and volumes of aerated and nonaerated parts of each pulmonary lobe using CT scans and compared data from healthy volunteers to ARDS patients. The results revealed considerable differences in lung morphology and distributions of gas within the lungs in ARDS patients as compared to healthy volunteers. Specifically, end-expiratory lung volumes and functional residual capacities were reduced in patients with ARDS, and this loss of gas volume was more pronounced in the lower as compared to the upper lobes. Over and above that, the authors classified distinct patient subgroups with either diffuse, lobar, or patchy attenuations that were associated with divergent outcomes in terms of mortality rates and their responsiveness to PEEP.

**Fig. 2:**
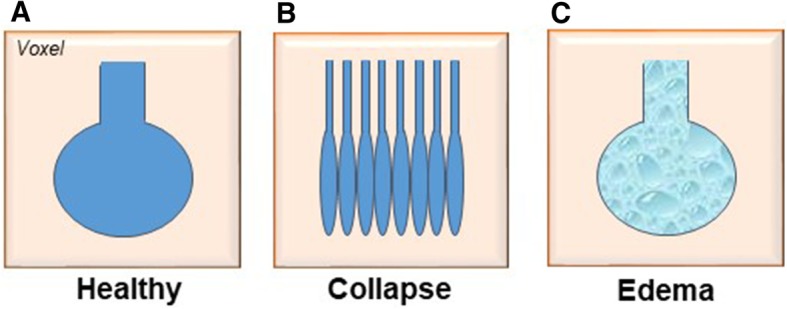
The concept of fixed CT pulmonary units under physiological (**a**) and pathophysiological conditions (**b**, **c**). The information given per fixed CT pulmonary units (voxel) is always stable, but the number of alveoli covered by a single voxel and the alveolar pathology might differ

Although the results of CT images fit well with experimental evidence on the usefulness but also the limitations of high PEEP ventilation, CT imaging has also been critiqued for its limited resolution, prohibiting observations at the alveolar level [65]. One major problem is to classify what a single voxel, defining the resolution of CT imaging, may include anatomically. At functional residual capacity in normal lungs, one voxel, considered as a fixed “CT pulmonary unit”, includes a defined number of the basic structures of a normal lung acinus, i.e., approximately 2000 alveoli [65]. However, under pathophysiological conditions such as complete or partial alveolar collapse, resulting in smaller volumes of individual alveoli, the number of alveoli contained in one single voxel may increase considerably. Conversely, in pulmonary edema, lung tissue will appear equally consolidated in CT scans, yet acinus dimensions remain unchanged, so that the actual fraction of the lung affected by lung edema will seem larger as in case of alveolar collapse (Fig. 2) [65]. The latter scenario also highlights a similar problem of CT imaging as previously discussed for IVM, namely the inability to differentiate between collapsed and fluid-filled lung areas. As a consequence of the “voxel problem,” CT imaging similarly cannot provide accurate data on tissue expansion, regional lung volumes, strain, or alveolar size, thus limiting its application with respect to the analysis of alveolar dynamics [45]. That notwithstanding, CT remains the gold standard imaging technology in patients with suspected lung disease.

### Electrical impedance tomography

Electrical impedance tomography (EIT) is a radiation-free functional imaging modality that was first introduced in the 1980s [76]. EIT records electrical currents to assess the conductivity distribution within the thorax from voltage measurements at its surface. Originally, this method had been invented at the beginning of the last century as a procedure exploring subterranean mineral deposits [77]. The clinical need for the assessment of the respiratory status and regional lung ventilation in mechanically ventilated patients has driven the rapid development of this technology and led to major advances of EIT in terms of its clinical use [78, 79]. In particular, the latest translational work by Amato and coworkers has received considerable attention from the scientific community. First, the authors demonstrated in several studies that utilization of EIT was capable to detect real-time dynamic changes in pulmonary ventilation and perfusion distributions in a pig model with one-lung ventilation [80–82]. Next, they used EIT in a clinical setting to individually identify the EIT-guided PEEP value producing the best compromise of lung collapse and hyperdistension in patients with abdominal surgery, hypothesizing that PEEP-EIT would vary among different patients and that it would reduce postoperative atelectasis [83]. The results corroborated that individual PEEP requirements vary widely among patients receiving protective tidal volumes during anesthesia. In addition, patients randomized to the EIT-guided strategy had less postoperative atelectasis (measured by CT) and improved intraoperative oxygenation and driving pressures, highlighting the critical role of sophisticated lung imaging techniques in clinical routine in general and for the delivery of personalized medicine in particular.

### Inhaled polarized gases

An emerging tool for studying lung micromechanics is the helium-3 (^3^He) lung morphometry, since it takes into account 3D structures, samples from all ventilated lung regions, and is spatially resolved [84]. Diffusion MRI experiments with hyperpolarized ^3^He measure anisotropic diffusion of ^3^He within alveolar ducts in images that cover the entire lung [84]. Based on the well-described relationships between anisotropic diffusion of hyperpolarized gases within acinar airways and the alveolar duct geometry [85, 86], values of alveolar duct radius and alveolar depth can be obtained within each voxel of the MR image. Resulting average values can be used to calculate the number of alveoli, alveoli surface area, and the volume of individual alveoli [84, 86]. Non-invasive studies using pulmonary MRI techniques have been used for the investigation of hyperpolarized ^3^He [85, 87–90] and xenon-129 (^129^Xe) [91, 92] to unravel lung microstructure. These experiments proved safe and suitable for the quantitative assessment of lung microstructure changes during emphysema when validated against histological measurements in clinical [86, 90] and preclinical settings [93–95]. However, the utilization of the technique is limited by its complexity and is cost-intensive due to the necessity of MR imaging.

### Theoretical models

In addition to experimental models and data from clinical patients, theoretical models have successfully advanced our understanding of alveolar dynamics and interdependence. This line of work was spearheaded by the landmark paper by Jere Mead and coworkers highlighting the interdependence of air-space distension and its relevance for uniform expansion of air spaces [96]. Subsequent work by Fung theoretically derived the stress-strain relationship of the lung parenchyma [97]. Schirrmann and coworkers used a basic simulation model for the biomechanical behavior of virtual single alveoli and simulated distinct in vivo conditions by modifying surface tension and tissue properties in order to compute parameterized P-V curves for different forms of lung injury [98]. The predicted data was in line with experimental data demonstrating that recruitment maneuvers and PEEP can stabilize the alveoli [50, 98]. More recently, simulation-based approaches have considered regions larger than individual alveoli, thus contributing to our understanding of lung stability [99–101]. In general, theoretical model present a versatile tool to obtain novel insights into alveolar mechanics and dynamics, especially when combined with experimental imaging approaches. Utilizing combined theoretical and experimental approaches may therefore present a promising avenue to further our in-depth understanding of alveolar (patho)-physiology.

## Alveolar dynamics in mechanically ventilated lungs

The application of these methodologies for the study of alveolar dynamics has fostered our insight into respiratory mechanics at the level of the individual gas exchange unit, yet it also generated a set of seemingly conflicting data and concepts which seem to be attributable, at least in part, not only to different methodologies but also to different interpretations of the observed phenomena.

### Alveolar dynamics in intact lungs

In the intact (i.e., healthy) lung, lung inflation has somewhat intuitively been attributed to expansion and contraction of individual alveoli. This concept was seemingly confirmed by histological analyses in rapidly frozen and fixed feline lungs demonstrating an increase in alveolar diameter from 128 ± 38 μm in deflation (*n* = 949 alveoli) to 168 ± 45 μm in inflation (*n* = 790 alveoli). Subsequent IVM analyses in ventilated rabbit lungs showed that the subpleural area of individual alveoli increases from 11,384 ± 2486 μm^2^ to 15,236 ± 3192 μm^2^ and 16,755 ± 3307 μm^2^ as airway pressure rises from 1 mmHg (in expiration) to 9 and 12 mmHg (in inspiration), respectively (unpublished data). These data were subsequently corroborated in ventilated mouse lungs by both IVM and OCT imaging [59]. Cyclic opening and collapse of alveoli, on the other hand, were not detected by either of the two imaging modalities [59]. Based on these data and theoretical considerations of alveolar interdependence [96] and opening pressures [98], a concept emerged in that alveoli are stable (i.e., permanently open) in intact mechanically ventilated lungs and that lung volume change occurs (predominantly) as a function of alveolar expansion and contraction.

However, the question of alveolar stability in healthy lungs has been a matter of controversy in the past. According to Laplace’s law

1 $$ \mathrm{Pressure}=2\times \mathrm{surface}\ \mathrm{tension}/\mathrm{radius} $$

intra-alveolar pressure is inversely related to the alveolar radius. As a result, smaller alveoli will tend to distribute their air to adjacent larger alveoli along the interalveolar pressure gradient until the smaller alveoli finally collapse. In healthy lungs, such a “heterogenization” of alveolar size is prevented by the stabilizing effect of pulmonary surfactant which increases in concentration in the alveolar lining fluid and therefore reduces surface tension approximately proportional to the decrease in alveolar radius [102–104].. Conversely, in injured lungs where the surfactant is degraded, deactivated, or insufficiently synthesized, alveolar clusters tend to become instable resulting in inhomogeneities of alveolar size and, ultimately, collapse of individual alveoli or even entire lung areas.

Contrary to the paradigm of alveolar stability in intact lungs, the group of Gary Nieman reported in a 1999 paper which drew considerable attention that lung volume change from deflation to inflation in the intact canine lung is not attributable to volume expansion of open alveoli, but predominantly results from cyclic recruitment and derecruitment of individual alveoli. This interpretation is based on the observation that the volume of open alveoli remained unchanged when lung volume was increased from 20 to 80% of total lung capacity, while the number of alveoli per alveolar surface area increased proportionally [44]. It goes without saying that the findings in the canine model are difficult to reconcile with the observations in mice and rabbit lungs obtained by IVM and OCT imaging, respectively. Species differences are an unlikely explanation given the overall similarities in alveolar anatomy and physiology between mammals. Higher gravitational gradients in the canine lung may affect alveolar dynamics but would be expected to result in less rather than more atelectasis in canine lungs in consideration of their higher gravitational level (lower West zone) as compared to mice given that both setups used upright microscopes, i.e., visualized the upper surface of the lung. Methods used for stabilization of the microscopic field—in the canine model, a coverslip was lowered onto the lung surface; in the murine model, lungs were freely moving under a transparent membrane glued to the chest wall—or open (canine) versus closed (murine) chest conditions may have contributed to these divergent findings; however, in the absence of a direct back-to-back comparison of both techniques by the same investigators, this remains speculative. Notably, the concept of cyclic opening and collapse as mechanism of lung volume change was recently supported by studies in healthy human volunteers using MRI of inhaled hyperpolarized gases (^3^He) suggesting that a 143 ± 18% increase in lung volume is associated with a 96 ± 9% increase in the total number of alveoli [84]. These findings seem to reinforce the recruitment/derecruitment hypothesis, yet it should be taken into account that the representation of alveolar recruitment by MRI diffusion imaging is problematic as areas with nonzero trapped gas volume during lung deflation will be considered as collapsed [62].

More importantly, a mechanism of volume change in the intact lung by continuous cyclic opening and collapse seems hard to reconcile with basic biomechanical principles as (a) based on Laplace’s law, the pressures required to open collapsed alveoli will markedly exceed those necessary to expand existing open alveoli [98], (b) repetitive opening and collapse would result in extremely high shear forces acting constantly upon the alveolar epithelium resulting in rapid epithelial injury as previously demonstrated for bronchial epithelium by Rolf Hubmayr’s group [105], and (c) repetitive opening and collapse would require high energy levels, resulting in rapid exhaustion of respiratory muscles and concomitant ergotrauma from energy expansion.

Of interest, a combination of alveolar expansion and alveolar recruitment has been proposed in a more recent confocal study on isolated lungs by Namati and coworkers with initial alveolar expansion and later recruitment, potentially via interalveolar pores of Kohn [106]. Notably, in this study, alveolar expansion was directly measured as > 100% change in mean alveolar chord length and a concomitant reduction in alveolar number per field view by > 75% when inflation pressure increased from 0 to 25 cmH_2_O. The notion of alveolar recruitment was derived from the subsequent observation that alveolar chord length tended to decrease (by approximately 10%) and the alveolar number increased (by approximately 25%) when inflation pressure was further increased from 25 to 35 cmH_2_O. Apart from the fact that the latter pressures are considerably supraphysiological in an isolated lung preparation, the interpretation of confocal alveolar images must be considered with due caution as vertical shifts of alveolar structures will generate the impression of significant volume changes or alveolar recruitment/derecruitment. Notably, a subsequent four-dimensional visualization of subpleural alveolar dynamics using optical frequency domain imaging and μ-CT in mechanically ventilated swine by the same author did not find indications of alveolar recruitment/derecruitment, but an almost ideal fit of the obtained data with a uniform expansion model [62]. Similarly, a recent study of alveolar dynamics using tracking X-ray microscopy in intact mice failed to detect any change in alveoli number or signs of recruitment/derecruitment, while alveolar size increased consistently, reproducibly, and reversibly from expiration to inspiration and vice versa [107]. Analogously, alveolar expansion by an average of 6.7% in diameter (equaling a 20% increase in volume assuming a spherical geometry) was recently detected by synchrotron X-ray imaging in live intact mice with a higher degree of inflation at the lung bases as compared to the apices [108].

Taken together, the majority of published data suggest that volume change in healthy lungs occurs primarily by alveolar expansion and contraction. Whether alveolar opening and collapse in healthy lungs exists remains still a matter of some controversy. As pointed out by Gil and Weibel already in 1972, alveolar opening and collapse will unquestionably occur along the usual static pressure-volume hysteresis ranging from collapse to near-maximal inflation [109]; however, for volume changes in the range of normal respiratory excursions, the contribution of opening and collapse is likely negligible.

### Alveolar dynamics in injured lungs

**Fig. 3:**
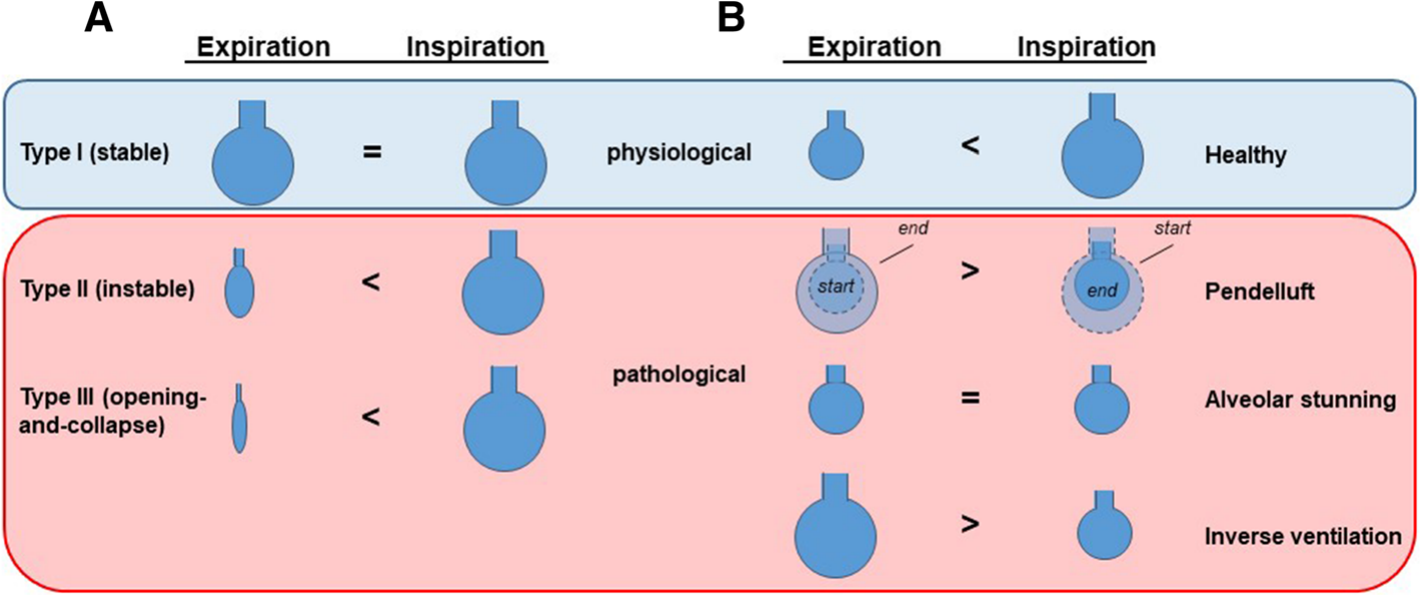
Concepts of alveolar dynamics in injured lungs. **a** Classification of alveolar dynamics according to [115]. Alveoli which do not change in size between end expiration and end inspiration are considered stable (physiologic, type I), those which increase in size during inspiration are considered instable (pathological, type II), or may even show alveolar collapse (pathological, type III). **b** Classification of alveolar dynamics according to [116]. Expansion of the alveoli during inspiration is considered as normal alveolar dynamics (physiological, healthy), paradoxical motion of alveoli during both the end-inspiratory and the end-expiratory plateau phase is classified as pendelluft (pathophysiologic), no change of alveolar size during end expiration or end inspiration is classified as alveolar stunning (pathophysiologic), and a decrease of alveolar size during end inspiration is defined as inverse ventilation (pathophysiological)

There is general consensus that alveolar dynamics change in injured lungs, a phenomenon that is often attributed to “alveolar instability.” However, the exact definition of “alveolar stability” and “instability” varies considerably between studies and research groups, which has slowed the emergence of a uniform concept (Fig. 3).

In 1997, Slutsky and colleagues introduced the concept of atelectrauma as an important pathomechanism of VILI in addition to volu- and barotrauma [110]. In mechanically ventilated rats, the authors demonstrated that the induction of VILI by ventilation with moderate tidal volumes was inversely related to PEEP, suggesting that repetitive opening and collapse of alveoli and small distal airways may have contributed to the aggravated injury at zero PEEP levels [110]. Based on these findings, alveolar instability is often used synonymous with alveolar opening and collapse, although it is worth to note that actual direct evidence for cyclic alveolar recruitment/derecruitment is missing in most studies of atelectrauma.

By the use of IVM, Nieman and coworkers have visualized alveolar dynamics in injured lungs in a sizable body of work; however, alveolar instability in these studies of lung injury following tween lavage was not primarily defined as opening and collapse, but by a ratio of total area change of alveoli within an area of observation between end expiration (E) and peak inspiration (I) with larger ratios of area change representing more pronounced alveolar instability [111–113]. According to the calculated ratio, alveolar dynamics were classified as type I (I-EΔ is almost zero), type II (increased I-EΔ), and type III (highly increased I-EΔ due to total alveolar collapse in end expiration [*E* = 0]) [114]. While type I was considered as “stable” alveoli, types II and III were interpreted as alveolar instability, with type III specifically reflecting cyclic opening and collapse. By the use of this classification, the authors demonstrated that high PEEP of 10 cm H_2_0 as compared to 5 cmH_2_O stabilizes alveoli [113]. Yet, no further alveolar stabilization was recognized with increasing PEEP above 9 cmH_2_O in spite of a continuous improvement in PaO_2_, which led the authors to conclude that alveolar stability/instability could not be identified simply by PaO_2_ [111]. In a recent comprehensive review, such principles of alveolar stability and instability were proposed as a future strategy to guide personalized mechanical ventilation in order to prevent VILI by stabilizing alveoli [25].

The notion that alveoli with I-EΔ ≈ 0 (type I), i.e., alveoli which do not change in size over the respiratory cycle, are stable, and alveoli with I-EΔ > 0 (type II), i.e., alveoli which expand with inspiration, are instable is based on the assumption that lung volume change in the healthy lung occurs exclusively by alveolar opening and collapse and that alveoli that are open at expiration will not change size during subsequent lung inflation [44]. If one considers, however, the alternative—and in the admittedly biased view of the authors of this article more probable—scenario that lung volume change in the healthy lung is primarily the result of alveolar expansion and contraction (vide supra), the conclusions from the above studies on alveolar stability actually reverse. Specifically, alveoli which do *not* change their size during inflation (type I) would then be considered “instable” or “altered,” while expanding alveoli (type II) would be considered “stable” or “physiological.” For example, with respect to the effects of PEEP, this understanding would imply that some alveoli are already fully expanded at high PEEP and, therefore, unable to expand further thus increasing the risk for overinflation of other airspaces—a very different interpretation from the original “alveolar stabilizing” effect of high PEEP [113]. Hence, our knowledge of alveolar dynamics in healthy lungs (or lack thereof) critically shapes our view of what is “physiological” or “pathological” alveolar behavior in injured lungs and, thus, which interventions may be advantageous or detrimental in a clinical setting.

In a series of IVM studies, our own group has also extensively characterized individual alveolar dynamics in murine models of acid-induced, transfusion-related, and surfactant-depleted lung injury [50, 58, 59]. Interestingly, while alveoli tended to expand uniformly and largely homogeneously in intact, healthy lungs [59], a number of impaired alveolar dynamics (termed “alveolar dyskinesias”) was observed in injured lungs. These dyskinesias ranged from alveolar pendelluft (i.e., a pendulum-like reciprocal air motion between a pair of adjoining alveoli) to alveolar stunning (i.e., alveoli with severely reduced tidal motion—similar to type I in the above definition) and inverse alveolar ventilation (paradoxical alveolar motion where alveolar volume is larger in the expiratory than in the inspiratory phase). Notably, these alveolar dyskinesias were directly associated with a locally impaired gas exchange, and restoration of “physiological” alveolar dynamics by short sighs also recovered local blood oxygenation [50]. These findings indicate that “stiff” type I alveoli represent in fact a pathological condition that contributes to impaired gas exchange and may aggravate the propagation of VILI.

**Fig. 4:**
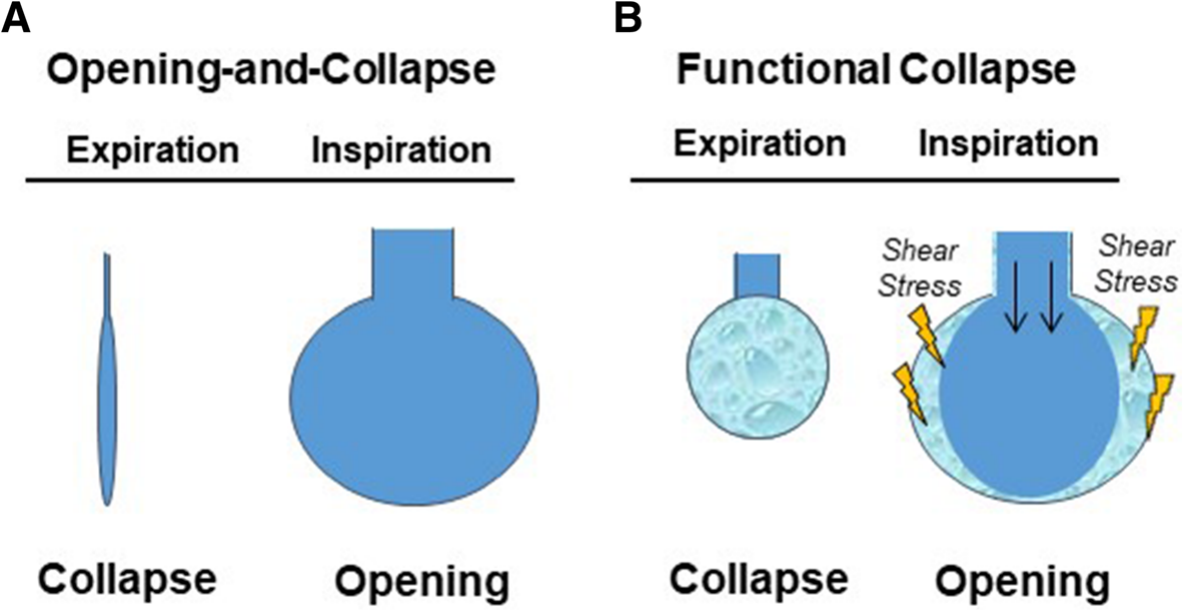
Concepts of structural and functional opening and collapse of individual alveoli in injured lungs. **a** The concept of structural opening and collapse hypothesizes a complete anatomical collapse of the alveolus during end expiration with a subsequent re-opening of the collapsed alveolus during end inspiration. **b** The concept of functional collapse hypothesizes fluid shifts into and out of the alveoli in a tidal fashion, causing high shear stress which damages the alveolar epithelium and impairs alveolar function

Importantly, despite the presence of various alveolar dyskinesias in a series of different models of lung injury, our group never observed cyclic opening and collapse of individual alveoli [50, 59], a finding that is in contrast to several other IVM studies [27, 113] and seemingly also to CT data in mechanically ventilated ARDS patients [68, 115]. This discrepancy is reminiscent of the scenario in healthy lungs; however, in addition to differences in species, methodologies, or hydrostatic pressures in the observational field, modes of lung injury (primarily surfactant depletion in studies showing alveolar opening and collapse) and severity of injury may come into play here. Over and above that, however, it is important to recall the specific limitations inherent to the various optical techniques utilized for the study of alveolar dynamics in vivo. Importantly, both IVM and CT imaging fail to distinguish between fluid-filled and collapsed alveoli. As increasing alveolar pressure will displace fluid from within the alveolus into larger airways and vice versa [116, 117], alveolar opening and collapse in injured lungs may in fact be a functional rather than a structural phenomenon. In other words, alveoli may become cyclically aerated and de-aerated by ventilation-dependent shifts of edema fluid in and out of injured alveoli in the absence of actual structural alveolar recruitment and derecruitment (Fig. 4). Notably, such a phenomenon would still be highly injurious to the alveolar epithelium due to the constant movement of an air-liquid interface along the alveolar epithelium [118]. The presence or absence of such a scenario would also be directly related to the extent of alveolar flooding in the area of observation and may as such be less prominent at the top surface of the lung visualized by IVM as compared to hypostatic lung regions imaged in patients in supine position. Functional rather than structural opening and collapse would be in line with simulation models predicting that structural alveolar collapse is unlikely [98] and that focal atelectasis can only occur if the entire lung is smaller than the resting volume [97], but also with studies using OCT imaging, notably the only optical method to discriminate between fluid-filled and collapsed alveoli [56], which failed to detect alveolar opening and collapse in injured lungs [59].

Importantly, the notion that structural opening and collapse may be a rare phenomenon in injured lungs and that the actual danger of atelectrauma lies more in the reduction of actual ventilated airspace (“baby lung concept”) rather than epithelial disruption by constant recruitment and derecruitment of alveoli is in agreement with a series of experimental studies which suggest that areas of atelectasis are per se only a mild promoter of lung injury. Notably, inflammatory injury markers are relatively low in animal models of lung atelectasis as compared to those of lung overdistension [119], and stereologic analyses from an in vivo small animal model of dorsal atelectasis and ventral aeration show that alveolar injury occurs predominantly in aerated, and not in atelectatic, regions [120]. The latter findings are consistent with clinical data obtained by CT imaging and positron emission tomography in ARDS patients, demonstrating that inflammation is largely restricted to the aerated lung [121]. Hence, atelectasis does not appear to contribute to local injury; rather, it appears to protect the local atelectatic regions and exposes instead the aerated lung [122].

### The clinical relevance of alveolar dynamics

In the past, analyses of lung inflation and deflation at the macroscale, or of the alveolar structure at the microscale, have generated important insights into the detrimental effects of mechanical ventilation and its underlying structural defects such as alveolar stress failure [123] with direct clinical implications. What has, however, been missing so far is an integrative understanding of the dynamic mechanics of the ventilated lung at the level of the individual alveolus. This information would not only link macroscale lung dynamics and microscale alveolar structure, but also serve to confirm—or refute—current concepts of mechanical ventilation which are commonly based on assumptions of alveolar dynamics as derived from indirect measurements, macroscopic imaging techniques, or downscaling from whole lung mechanics.

While translation of alveolar dynamics into clinical practice has been scant in the past due to (a) the complexity and challenges involved in the analysis of alveolar dynamics outlined above, (b) the relative paucity of actual data on alveolar dynamics, and (c) the relative lack of tools to directly image alveolar dynamics in humans, two examples should be highlighted here: first, by IVM of alveolar dynamics in five anesthetized rats subjected to saline-lavage lung injury, DiRocco, Carney, and Nieman showed that individual alveolar recruitment does not correlate with the lower inflection point of the pressure-volume curve on inflation, yet alveolar derecruitment correlated with the upper inflection point during lung deflation [27]. These findings have direct clinical implications, as they suggest that the upper rather than the traditionally used lower inflection point may be a better indicator for the optimal setting of PEEP [124]. Second, intravital analysis of alveolar dynamics from our own group identified pendelluft as an early form of alveolar dyskinesia which can be reverted by individual sighs, a finding that (a) provides a mechanistic explanation for a series of preclinical studies demonstrating the benefits of sighs (in the absence of subsequent PEEP elevation) [125, 126] and (b) has been successfully translated into the clinical scenario [127, 128]. Along similar lines, it can be expected that further in-depth understanding of alveolar dynamics such as the extent and relevance of alveolar opening and collapse, the impact of respiratory rate, inspiratory flow, inspiration-to-expiration ratio, or the role of alveolar interdependence may provide novel insights to inform optimized and—ideally—personalized ventilation strategies.

## Conclusion

Taken together, there is general agreement that lung injury results in alveolar instability and alveolar dyskinesias which may further promote and aggravate VILI; yet, the classification of impaired alveolar dynamics depends critically upon our definition of healthy alveolar mechanics, for which there is still a lack of consensus. Particular areas of controversy relate to the question what constitutes a “stable” versus “instable” alveolus, whether cyclic opening and collapse occurs at the structural or functional level, and the extent to which these phenomena are in fact pathophysiologically and, thus, clinically relevant.

Given the enormous implications of ventilation strategies in the clinical setting and ultimately, for patient outcome, the apparent lack of insight and consensus must be concerning. While high-end imaging techniques and infrastructure have allowed for unprecedented insight into the ventilated lung, different approaches and methodologies tend to yield seemingly contradictory results. While seeing commonly leads to believing, it may at times also be misleading. In order to advance the field of knowledge, joint task forces aiming to reconcile seemingly discrepant findings, and—even better—combined research efforts merging key technologies in identical models would seem the most promising road to advance our knowledge and reach a higher level of evidence and consensus. Such a combined approach would allow to minimize the limitations inherent in each individual imaging technique and, thus, not only to provide conclusive insights into lung physiology, but ultimately to guide patient therapy.

## Abbreviations

^129^Xe: 129-Xenon
3D: Three-dimensional
3He: Helium-3
ARDS: Acute respiratory distress syndrome
bw: Body weight
CT: Computed tomography
E: End expiration
I: Peak inspiration
IVM: Intravital microscopy
OCT: Optical coherence tomography
PEEP: Positive end-expiratory pressures
P-V: Pressure-volume
VALI: Ventilator-associated lung injury
VILI: Ventilator-induced lung injury
